# Regulatory T Cells in the Tumor Microenvironment and Cancer Progression: Role and Therapeutic Targeting

**DOI:** 10.3390/vaccines4030028

**Published:** 2016-08-06

**Authors:** Belal Chaudhary, Eyad Elkord

**Affiliations:** 1 Cancer Research UK Cambridge Institute, University of Cambridge, Cambridge CB2 0RE, UK; belal.chaudhary@cantab.net; 2 Cancer Center, Qatar Biomedical Research Institute and College of Science and Engineering, Hamad Bin Khalifa University, Qatar Foundation, Doha 5825, Qatar; 3 College of Medicine and Health Sciences, United Arab Emirates University, Al Ain 17666, UAE; 4 Institute of Cancer Sciences, University of Manchester, Manchester M20 4BX, UK; 5 Biomedical Research Centre, School of Environment and Life Sciences, University of Salford, Salford M5 4WT, UK

**Keywords:** regulatory T cells, tumor microenvironment, cancer progression, therapeutic targeting, tumor-infiltrating lymphocytes

## Abstract

Recent years have seen significant efforts in understanding and modulating the immune response in cancer. In this context, immunosuppressive cells, including regulatory T cells (Tregs) and myeloid-derived suppressor cells (MDSCs), have come under intense investigation for their proposed roles in suppressing tumor-specific immune responses and establishing an immunosuppressive tumor microenvironment, thus enabling tumor immune evasion. Additionally, recent evidence indicates that Tregs comprise diverse and heterogeneous subsets; phenotypically and functionally distinct subsets of tumor-infiltrating Tregs could contribute differently to cancer prognosis and clinical outcomes. Understanding Treg biology in the setting of cancer, and specifically the tumor microenvironment, is important for designing effective cancer therapies. In this review, we critically examine the role of Tregs in the tumor microenvironment and in cancer progression focusing on human studies. We also discuss the impact of current therapeutic modalities on Treg biology and the therapeutic opportunities for targeting Tregs to enhance anti-tumor immune responses and clinical benefits.

## 1. Introduction

Regulatory T cells (Tregs) comprise diverse subsets of immunosuppressive cells that play critical roles in maintaining immune homeostasis and self-tolerance. They are also involved in controlling autoimmunity, infection, graft-versus-host disease, inflammation, fetal-maternal tolerance, and tumor immunity [1].

Tregs are broadly divided by lineage into thymic-derived tTregs, autoreactive T cells selected by high avidity interaction with self-antigens in the thymus, and peripheral pTregs, induced from naïve CD4^+^ T cells by sub-optimal antigen presentation in the periphery [2]. tTregs are crucial for preserving self-tolerance and preventing autoimmunity, while pTregs maintain peripheral tolerance at mucosal interfaces and in response to external antigens [1,3]. Both subsets of Tregs have traditionally been defined by expression of the Forkhead Box P3 (FoxP3) transcription factor––a “master regulator” of the suppressive lineage—and the IL-2 receptor α chain (CD25) [1,4]. pTregs additionally comprise two FoxP3^−^ subsets with important roles in oral tolerance: Tr1 and Th3 cells [5].

Tregs may exert their suppressive activity via a number of contact-dependent and independent mechanisms, as reviewed extensively [6,7]. Briefly, these include:

- Suppressive cytokines (TGF-β, IL-10, IL-35)
- Immune checkpoints and inhibitory receptors (CTLA-4, PD-1, LAG-3, TIM-3, ICOS, TIGIT, IDO)
- Direct cytotoxicity (perforin/granzyme-mediated)
- Metabolic disruption of T effector cell activity (IL-2 consumption)
- Induction of tolerogenic DCs, which promote T cell exhaustion and expansion

Tregs exhibit considerable phenotypic and functional specialization according to tissue localization, disease state, activation and differentiation status [8,9,10,11]. These subsets play differing roles in disease and health, and may be endowed with one or more of these suppressive mechanisms [12,13].

## 2. Tregs in Cancer

In cancers, Tregs are able to suppress anti-tumor immune responses and contribute to the development of an immunosuppressive tumor microenvironment (TME), thus promoting immune evasion and cancer progression [14,15].

Tregs have been extensively characterized in the peripheral blood and immune infiltrates of different cancers [15,16]. An accumulation of FoxP3^+^ Tregs and, in particular, a higher Treg : T effector cell (Teff) ratio within tumor tissue is associated with worse prognoses in many cancers, including ovarian cancer [17,18], pancreatic ductal adenocarcinoma [19,20], lung cancer [21], glioblastoma [22], non-Hodgkin’s lymphoma [23], melanoma and other malignancies [24,25]. The role of Tregs in immune escape is supported by clinical studies, and numerous in vitro studies, where Treg depletion released anti-tumor immunity. For example, transient Treg depletion induced regression of metastatic lesions in advanced stage melanoma patients [26]. In breast cancer patients undergoing tumor resection and radiotherapy, Treg depletion prior to treatment is associated with an anti-tumor immune response and improved clinical outcomes [27]. Additionally, Treg depletion followed by cancer antigen vaccination generated effective anti-tumor CD4^+^ and CD8^+^ T-cell responses in metastatic breast cancer patients [28].

It should also be noted that Treg infiltration and accumulation can correlate with a positive prognosis in certain malignancies, including colorectal and gastric cancers [29,30]. In these cancers, Tregs are proposed to play a protective role by controlling inflammation associated with neoplastic transformation and cancer progression. This topic was recently reviewed [31] and will be briefly discussed.

Tregs are able to accumulate within the TME via several mechanisms [32]. These are summarized below:

- *Recruitment:* Tregs are recruited into tumors in response to chemokines secreted by tumor cells and innate immune cells; key chemokine-chemokine receptor combinations include CCL17/22-CCR4, CCL5-CCR5, CCL28-CCR10 and CXCL9/10/11-CXCR3.
- *Expansion:* Tregs can be expanded in situ, and proliferate efficiently in response to tumor-derived factors (TGF-β, IL-10) within the TME.
- *Conversion:* Generation of suppressive Tregs from non-suppressive CD25^−^ conventional T cells (Tconv) driven by tumor-derived transforming growth factor-beta (TGF-β) and adenosine; this has mainly been studied in murine models and the contribution of Treg induction to Treg accumulation within the TME in human cancer remains to be confirmed.

Further mechanisms of Treg recruitment and generation are still being uncovered. For example, sphingosine 1-phosphate (S1P)—a bioactive lipid mediator involved in angiogenesis and inflammation—is important for immune cell trafficking and is able to restrain Treg development in the periphery [33]. In pre-clinical models, S1P receptor 1 (S1PR1) signaling was necessary for Treg accumulation within the TME, acting via the JAK/STAT-3 signaling pathway [34]. The importance of S1P/S1P receptor signaling for the immune response in human cancer remains to be confirmed.

As highlighted by the variable impact of Tregs in different cancers, the role of Tregs in cancer is multi-faceted and is influenced significantly by cancer type, stage and location, in addition to the unique immune landscape and TME of each cancer [24,25,35,36]. This review focuses on the role of Tregs as suppressors of anti-tumor immune responses, and specifically on their roles within the TME.

### 2.1. Immunosuppressive Roles of Tumor-Infiltrating Tregs in Cancer

Tumor-infiltrating (TI) Tregs play direct roles in promoting immune evasion and the development of a pro-tumorigenic TME. They exhibit distinct phenotypic and functional profiles, upregulating markers associated with activation and enhanced suppressive activity. These include immune checkpoint molecules, cytotoxic T-lymphocyte associated protein 4 (CTLA-4), T-cell immunoglobulin and mucin-domain containing-3 (TIM-3/HAVCR2), lymphocyte activation gene-3 (LAG-3), programmed-death 1 (PD-1), inducible T-cell co-stimulator (ICOS), and glucocorticoid-induced TNFR family related gene (GITR); and T cell activation markers, CD25 and CD69 [37,38,39,40,41,42,43,44,45].

Numerous studies have identified suppressive Treg subsets in the peripheral blood of cancer patients. However, direct insights into the suppressive roles of Tregs within the TME are limited. FoxP3^+/−^ TI Treg subsets isolated from primary tumors of colorectal cancer (CRC) patients exerted a potent suppressive activity mediated by TGF-β and IL-10, and also upregulated CTLA-4 and ICOS [44]. In hepatocellular carcinoma (HCC) and pancreatic cancer patients, two distinct FoxP3^+/−^ TI Treg subsets exhibiting differential expression patterns of CTLA-4, PD-1, CD25 and CD69 were identified in tumor-infiltrating lymphocyte (TIL) populations. These TI Tregs suppressed the activity of autologous CD4^+^ T cells and gamma delta (γδ) T cells via secretion of TGF-β and IL-10 [37,46,47]. In another HCC study, FoxP3^−^CD69^+^CTLA-4^+^PD-1^+^ Tregs were enriched within the TME where they comprised over 60% of the CD4^+^ TIL populations and suppressed autologous Teff via membrane-bound TGF-β [43]. FoxP3^+^ TI Tregs from gastric cancer patients were shown to exert suppressive activity via production of cyclooxygenase-2 (COX-2) and prostaglandin E-2 (PGE-2) [48]. Other groups have isolated highly suppressive FoxP3^+^ Tregs expressing CTLA-4, GITR and TIM-3 from immune infiltrates of HCC, CRC, cervical and ovarian carcinomas [17,42,49,50].

These studies highlight the varied suppressive functionality and phenotype of TI Tregs. A number of the markers expressed on TI Treg subsets are directly involved in suppressive function. Inhibitory immune checkpoint molecules, such as CTLA-4, PD-1, LAG-3 and TIM-3, act to dampen immune responses and prevent excessive T cell activation during physiological immune responses. CTLA-4 promotes T cell suppression by preferentially binding with CD80/86 signaling molecules over CD28, effectively blocking CD28 co-stimulatory signals required for T cell activation. Similarly, LAG-3, TIM-3 and PD-1 are inhibitory receptors that negatively regulate Teff and CD8^+^ cytotoxic lymphocyte (CTL) function, as well as potentially promoting Treg generation and function [51,52].

The expression of other functional markers may also imply in vivo suppressive activity of Tregs. Co-expression of GARP/LAP on Tregs infers the presence of membrane-bound latent TGF-β complexes. Latency-associated peptide (LAP) sequesters TGF-β in inactive latent TGF-β complexes, which are anchored to the surface of T cells by transmembrane glycoprotein A repetitions predominant (GARP) [53,54]. Latent TGF-β complexes can be cleaved to release active TGF-β in response to a variety of signals. Highly suppressive LAP^+^ and GARP/LAP co-expressing Tregs have been identified in TILs of CRC patients and the peripheral blood of pancreatic, CRC and anti-CTLA-4-treated bladder cancer patients, where their suppressive activity was mediated by TGF-β and IL-10 [44,55,56,57]. Similarly, the ectonucleotidases, CD39/CD73, act synergistically to generate immunosuppressive adenosine from exogenous ATP; a key suppressive pathway within the TME [58,59]. CD39 and CD73 are rarely co-expressed on human Tregs; however, CD39 has been shown to be highly expressed on intra-tumoral Tregs in colon and head & neck cancers (HNC) [39,59,60,61]. Interestingly, CD39^+^ Tregs can interact directly with CD73^+^ cells or CD73^+^ exosomes derived from the TME to produce adenosine [60,62].

#### Enhanced Suppression in the TME

TI Tregs exhibit enhanced suppressive capacity compared to Tregs isolated from peripheral blood and healthy tissue [39,42,50,63]. This may in part be due to increased Treg activation within the TME, where Tregs are exposed to tumor-associated antigens (TAA) and neoepitopes [32]. TAAs are derived from self-antigens which Tregs detect with a high affinity compared to Teff, thus enabling their preferential activation. Tregs can be divided into three distinct subsets based on their activation and differentiation status, as described by Miyara et al. [8]:

1. CD45RA^−^FoxP3^hi^ activated “effector” Tregs
2. CD45RA^+^FoxP3^lo^ resting Tregs
3. cytokine-secreting CD45RA^−^FoxP3^lo^ non-suppressive T cells, or “non-Tregs”

“Effector” Tregs (eTregs) represent a terminally differentiated and highly suppressive Treg subset. eTregs have been shown to predominate within TIL populations in CRC, lung cancers and melanomas [40,41,45,56]. In CRC patients, it has recently been shown that tumor infiltration by FoxP3^hi^ eTregs was associated with poorer prognosis, compared to tumors infiltrated by non-suppressive FoxP3^lo^ T cells [64]. Depletion of circulating CCR4^+^ eTregs by an anti-CCR4 mAb restored antigen-specific CTL responses in an adult T-cell leukemia-lymphoma patient [41]. Activated Tregs have also been shown to upregulate expression of functionally suppressive molecules (GARP/LAP, CD39/CD73) and immune checkpoints (CTLA-4, TIM-3, GITR, PD-1, LAG-3), thus potentiating greater suppressive activity.

The selective accumulation of eTregs within tumor tissue, but not peripheral blood, suggests eTregs are preferentially recruited and/or activated within the TME [40,41,45,56]. The exact mechanism by which highly suppressive and activated eTregs might accumulate within the TME is still an open question and may vary by cancer, although it likely involves chemotactic migration. As studied extensively, Tregs isolated from healthy donors and cancer patients can express a plethora of chemokine receptors (CCR2-9, CXCR3/4) and migrate efficiently in vitro in response to tumor-derived chemokines, typically secreted by cancer cells or innate immune cells [32,45,65]. The CCL17/22-CCR4 axis has been shown to be particularly important in lymphomas, lung, breast, ovarian, gastric and prostate cancers [32,65]. Additionally, Tregs have been reported to proliferate efficiently in vivo in the peripheral blood and particularly within the tumor tissues of CRC and metastatic prostate cancer patients [44,66]. In CRC, a greater proportion of CD25^+^FoxP3^+^ TI Tregs expressed Ki67, compared with Tregs from tumor-free liver and peripheral blood, indicating efficient in situ proliferation [42]. In comparison, Tregs isolated from tumor tissue, tumor-free liver and peripheral blood of HCC patients all expressed significantly lower levels of Ki67 than TI Tregs from CRC patients [42]. This again highlights the differences in Treg biology in different cancers.

Understanding the most relevant Treg mechanisms or markers within the TME is important for designing Treg-targeted immunotherapies. For example, CCR4-targetted antibodies (Abs) have shown promising results, inducing effective depletion of FoxP3^+^ Tregs both in vitro and in vivo [41,45,67,68,69,70].

### 2.2. TME-Treg Crosstalk

The TME is a critical contributing factor to immune evasion and the efficacy of immunotherapeutic approaches in cancer. Tumor cells, tumor-infiltrating immune cells, stromal cells, and other infiltrating cell subsets work in concert to establish a TME that is tolerogenic, hypoxic, rich in pro-angiogenic growth factors and highly immunosuppressive [71,72]. Cell-cell crosstalk is important for normal physiological function and maintenance of homeostasis. In cancer, Tregs interact with infiltrating immune cell subsets, stromal cells and tumor cells within the TME to enhance Treg generation and suppressive function, as recently reviewed [73]. Herein, we briefly summarize the interactions between Tregs and different components of the TME.

*Natural Killer (NK) cells:* Studies of NK cell-Treg interactions in human cancer are limited [74]. Circulating Tregs isolated from healthy donors and gastrointestinal stromal tumor patients are able to suppress NK cells via membrane-bound TGF-β, downregulating expression of the activating NK cell receptor (NKG2D) [75]. Similar findings were reported in cervical carcinomas where TI and circulating Tregs potently suppressed NK cell activity in vitro [76].

*Myeloid-derived Suppressor cells (MDSCs):* MDSCs are immunosuppressive cells that are critical to promoting tumor-immune evasion, in collaboration with Tregs [77,78]. Both Tregs and MDSCs are able to induce generation and enhance the suppressive activity of the other via various signaling pathways, including inhibitory programmed death ligand-1 (PD-L1/B7-H1) signaling [79,80]. Their interactions in cancer have recently been reviewed in detail [73,81].

*Antigen-presenting cells (APCs):* Similar to other lymphocytes, Tregs rely on APCs such as dendritic cells (DCs) and macrophages for antigen presentation and T cell activation. DCs and Tregs exhibit extensive bi-directional cross-talk, influencing the immune response both in physiological and pathological settings. Within the TME, naïve tolerogenic DCs and plasmacytoid dendritic cells (pDCs) promote Treg function and generation [82,83]. Through a number of mechanisms—including production of TGF-β and IL-10, and upregulation of inhibitory B7-H3/4 molecules on DCs—Tregs are able to influence DC maturation, directing them towards a tolerogenic phenotype and impairing their ability to activate Teff and CTLs.

*Stromal cells:* The stroma is intimately involved in cancer initiation, progression and metastasis. However, Treg–stromal interactions in humans are not well understood. Initial in vitro work suggests that stromal cells may be important in recruiting and generating Tregs at tumor sites, possibly through cell contact-dependent mechanisms and secretion of soluble mediators including TGF-β, PGE-2, and indoleamine 2,3-dioxygenase (IDO) [84].

*Endothelial cells (ECs):* ECs and lymphatic endothelial cells (LECs) typically come into contact with antigens, cytokine and migrating immune cells at an early stage, and play important roles in controlling the immune response and immune cell trafficking. ECs have been shown to enhance Treg migration, and to induce Treg generation under inflammatory conditions [85,86,87]. Tregs are also able to interact with ECs to restrain Teff migration, by impairing EC selectin expression and adhesion to Teff [88].

*Tumor angiogenesis:* Tumor angiogenesis is a key step in cancer development and progression, driven by pro-angiogenic growth factors such as vascular endothelial growth factor-A (VEGF-A). Intra-tumoral accumulation of FoxP3^+^ Tregs is associated with increased tumor vasculature density in endometrial cancers [89]. In an in vitro model of ovarian cancer, Tregs conditioned under hypoxic conditions, similar to the in vivo TME, secreted significantly higher levels of VEGF-A compared to normoxic conditions and efficiently induced endothelial-tube formation in vitro [90].

*Tumor-derived exosomes (TEX):* TEX are a newly identified mechanism by which tumor cells may enhance Treg function. TEX deliver tumor-derived factors which enhance Treg suppressive activity, lineage stability and resistance to apoptosis [60,91]. Interestingly, TEX have also been shown to modulate adenosine pathway-related gene expression in Tregs, potentially promoting deaminase activity [91].

### 2.3. Protective Role of Tregs in Inflammation/Cancer

Recent studies show that an intra-tumoral accumulation of FoxP3^+^ Tregs is associated with positive prognoses in certain malignancies, including CRC, HNC, and esophageal cancers [25,31]. In these cancers, Tregs are proposed to play protective roles by controlling tumor-promoting inflammation [31,92,93].

Inflammation contributes both to cancer initiation and progression by promoting genomic instability, neoplastic transformation, tumor metastasis, tumor angiogenesis, and survival and proliferation of malignant cancer cells [94]. Inflammation has been linked to increased incidence of malignancies of the gut, airway and mucosal interfaces; these are “inflammation-prone” sites which are continually exposed to microbial and foreign antigen challenge and are particularly sensitive to perturbations in immune homeostasis or local microbiota [36,95]. The initial inflammatory insult contributing to neoplastic transformation may have different sources: (i) chronic viral infections such as human papilloma virus (HPV) in head and neck cancers, or hepatitis B/C virus (HBV/HCV) in HCC; (ii) exposure to tobacco and other carcinogens in lung cancers; or (iii) increased penetration of the mucosal barrier by commensal bacteria prior to development of CRC [36]. Tregs are critical to maintaining tolerance in the airways and at gut and mucosal interfaces [1,5,96]. Given the link between inflammation and cancer, it is feasible that Tregs play protective roles prior to cancer initiation in “inflammation-prone” cancers [97].

There are a number of open questions with regards the protective role of Tregs in cancer. Are the Treg subsets involved in inhibiting anti-tumor immunity distinct from those involved in controlling potentially tumorigenic inflammation? Following tumor establishment, can “protective Tregs” be co-opted by tumors and undergo a switch to a pro-tumorigenic role? For example, Tr1 cells, critical mediators of oral tolerance, have been shown to accumulate in the peripheral blood of HNC patients with advanced disease stage and in patients showing no sign of active disease following successful therapy [98]. An accumulation of highly suppressive and activated FoxP3+ TI Tregs in the tumor tissue of CRC patients has also been reported to correlate with tumor progression [40,99].

It should be noted that the majority of clinical studies currently utilize FoxP3 and CD25 as Treg markers. While FoxP3^+^CD25^hi^ T cells include significant populations of suppressive Tregs, both CD25 and FoxP3 can be highly upregulated on non-suppressive T cells during activation or inflammation [100,101]. It is critical that the suppressive lineage of lymphocytes identified as Tregs is confirmed either functionally in suppression assays or by utilizing Treg markers relevant to the tissue context [102]. A number of reviews discussing the role of Tregs in colorectal cancer have highlighted this issue [31,58].

### 2.4. Treg Subsets and Heterogeneity

Tregs comprise diverse and heterogeneous subsets displaying tissue- and disease-specific phenotypic and functional features. Defining the different subsets and understanding their roles in immune evasion is crucial for designing effective immunotherapies. Herein, we briefly describe the different Treg subsets.

*pTregs and tTregs:* The exact composition of TI Treg subsets has been a subject of debate, focusing mainly on FoxP3-expressing tTregs and pTregs [92,103]. Efforts to define the specific roles of pTregs and tTregs in cancer have been hindered in part due to the lack of effective Treg markers. Neuropilin 1 (NRP1) and the Ikaros zinc finger transcription factor, Helios, enable identification of tTregs and extra-thymically induced pTregs in mice but not in humans, although they can be expressed on highly suppressive Treg subsets [16,57,92,103,104]. Helios, in particular, may represent an important Treg marker although its exact role in human Tregs remains to be confirmed [57].

*Tr1 cells:* Highly suppressive Tr1 cells have been characterized in Hodgkin’s lymphoma, head and neck squamous cell carcinoma (HNSCC), HCC and CRC [44,83,98,105]. Tr1 cell generation from naïve CD4^+^ T cell precursors is promoted at tumor sites mainly through the action of immature DCs or tolerogenic pDCs, as shown by in vitro models of HNSCC, HCC and liver metastases of CRC [83,98]. In a CRC study, Tr1 cells were shown to comprise up to 30% of the tumor-infiltrating lymphocyte subsets [44]. These TI Tr1 cells produced IL-10 and TGF-β, and exhibited an in vitro suppressive activity up to 50 times more potent than autologous FoxP3^+^ Tregs [44]. Although Tr1 cells represent a highly suppressive Treg subset, their clinical impact in cancer is still uncertain. An increase in the Tr1 cell : FoxP3^+^ tTreg ratio was associated with longer survival in a small clinical study of four recurrent ovarian cancer patients undergoing adoptive T cell transfer with autologous IL-10 and interferon gamma (IFN-γ)-producing Teff [106].

*Th3 cells:* Similar to Tr1 cells, Th3 cells are important for maintaining oral tolerance. While they have been studied to a certain extent in murine models, their contribution and relevance to human cancers is not clear [5].

Interestingly, Tregs are also able to partially co-opt the transcriptional profile of T helper (Th) cell subsets in order to home into specific sites within the body while maintaining their suppressive lineage [9,107]. This is thought to occur in response to inflammatory or environmental signals. For example, in an ovarian carcinoma study, the majority of FoxP3^+^ TI Tregs upregulated the Th1 master transcription factor, T-bet, and expressed CXCR3 enabling them to migrate in response to CXCL10 [108]. These FoxP3^+^CXCR3^+^ were suppressive ex vivo, co-expressed Helios and, intriguingly, their numbers matched those of CXCR3^+^ Th1 cells, suggesting both may have been recruited into the TME down a similar CXCL10 chemotactic gradient. In addition to these subsets, specialized tissue-resident Tregs can also be recruited by tumors, although their exact contribution to tumor immunity is not known [93].

### 2.5. Antigen Specificity of Tregs

Tumors are thought to present neo-epitopes that may preferentially activate Tregs and promote tumor-specific immune suppression within the TME [109,110]. Antigen (Ag)-specific Tregs have been isolated from the peripheral blood and TILs of CRC, pancreatic cancer, bladder cancer, melanoma and other cancers [46,99,111,112,113]. Suppressive Ag-specific Tregs have also been induced in vitro and in vivo in melanoma patients following peptide stimulation or immunization [111,112]. In pancreatic cancer patients, FoxP3^+^IL-10^+^TGF-β^+^ TI Tregs comprised 12% of the ENO1-specific CD4^+^ T cell population and specifically inhibited the proliferation of autologous ENO1-specific Teff [46]. ENO1-specific Tregs were able to interact with ENO1 at significantly lower concentrations of antigen than ENO1-specific Teff, suggesting that the Ag-specific Treg TCR had a higher affinity for ENO1 [46]. In CRC patients undergoing resection, the presence of suppressive Tregs specific for the CRC-associated antigens, CEA and 5T4, was correlated with tumor recurrence and relapse [99]. A recent bladder carcinoma study identified a number of TAAs that Tregs were specific for; interestingly, Teff specific for the same TAAs did not co-occur in these patients [113]. Similarly, in ovarian cancer, Tregs were shown to co-exist with NY-ESO-1-specific Teff within tumor tissue, but were not specific for NY-ESO-1 [114].

The full extent and relevance of Ag-specific Treg responses to clinical outcomes is not yet clear. Tregs are able to exert non-specific “bystander tolerance” following in vitro stimulation. Further studies are required to confirm the importance of Treg Ag specificity in immune evasion.

### 2.6. Tregs by Cancer Stage

Tregs have been studied extensively in established tumors where an accumulation of functionally suppressive Tregs tends to correlate with advancing tumor stage, and is accompanied by concomitant Teff and CTL impairment. The immune landscape within tumors, however, varies greatly by stage and the contribution of Tregs during other phases of cancer progression is less clear [115]. Herein, we briefly discuss possible roles of Tregs during cancer initiation and establishment, metastasis, remission and recurrence.

#### 2.6.1. Cancer Initiation and Establishment

During early neoplastic events, innate immune cells are typically “first on the scene”. In response to chemokines secreted by tumor cells and innate immune cells, Tregs are recruited to cancerous lesions or tumor-draining lymph nodes (TDLN) where they are chronically exposed to and activated by TAAs and tumor-derived factors [116,117]. The net result is an accumulation of suppressive Tregs in TDLN and tumor tissue compared to functional Teff and CTLs. As discussed earlier, these activated TI Tregs downregulate the anti-tumor activity of Teff, CTLs, NK cells and DCs, and secrete immunosuppressive molecules, setting the scene for tumor progression and growth. The question remains—how critical are Tregs to the initiation and establishment of cancers along with MDSCs, macrophages, mast cells and other innate immune cells? Do perturbations in immune homeostasis contribute to neoplastic transformation? In inflammation-associated cancers, the contribution is clear. However, in other malignancies, the exact role of the immune system likely depends on specific tumor “immunogenicity” and immunobiology.

The role of Tregs in the early immune response to cancer has primarily been investigated in murine studies, given the technical difficulties with studying Treg biology prior to cancer detection in humans [118]. Interesting parallels have been drawn between the early immune response, and subsequent induction of immune tolerance, in human pregnancy and cancer [118]. The immune privilege offered to developing neoplasms by Tregs may mirror that of a developing embryo, representing a highly effective and evolutionarily conserved immune tolerance mechanism that is co-opted by tumors for their own benefit. An exception to this model, as noted earlier, may be at gut, airway and mucosal interfaces where Tregs are thought to play a protective role, controlling cancer-associated inflammation.

#### 2.6.2. Tumor Metastasis

The potential roles of Tregs in promoting and establishing metastatic sites were discussed in an extensive review [119]. Tregs have been shown to infiltrate metastatic sites in various cancers, where they often also predict worse outcomes. At these metastatic sites, Tregs are proposed to play similar roles as within the TME, promoting immune tolerance and immune evasion. A number of questions remain unanswered in humans; do Tregs contribute directly to “conditioning” of pre-metastatic niches? Which mechanisms of Treg recruitment and suppression are most relevant for metastases?

Pre-clinical murine models show that Tregs are important for establishing metastatic sites following dissemination of tumor cells, highlighting in particular the role of receptor activator of nuclear factor kappa-B ligand (RANKL) on Tregs in directly promoting RANK^+^ breast cancer invasion [120]. Intriguingly, human CD25^+^CD127^lo^ Tregs isolated from the peripheral blood of HCC patients were recently shown to enhance proliferation of the HepG2 cell line in vitro via upregulation of the RANK-RANKL pathway [121]. Further investigations into the mechanisms of Treg recruitment and their roles at metastatic sites are warranted.

#### 2.6.3. Remission/Recurrence

As discussed earlier, the critical suppressive role of Tregs in inhibiting anti-tumor immune responses in many cancers is now well-accepted. A natural follow-on question is whether Tregs are directly involved in mediating tumor recurrence or remission. The presence of suppressive Tregs prior to resection, chemotherapies or radiotherapies has been shown to predict tumor recurrence and worse clinical outcomes; whether this link indicates a causal or correlative relationship, however, is not clear and must be confirmed in vivo [122]. Tumors often display a “T cell inflamed” phenotype characterized by significant T cell infiltration and reliance on immunosuppressive networks (Tregs, IDO, adenosine) to evade anti-tumor immune responses; in these tumors, Tregs might play a more central role in promoting tumor remission or recurrence [123]. In contrast, “non-T cell inflamed” tumors exclude immune cells, have denser stroma and are often infiltrated by immunosuppressive MDSCs [123].

## 3. Clinical Evidence for Treg Therapeutic Targeting

**Figure 1:**
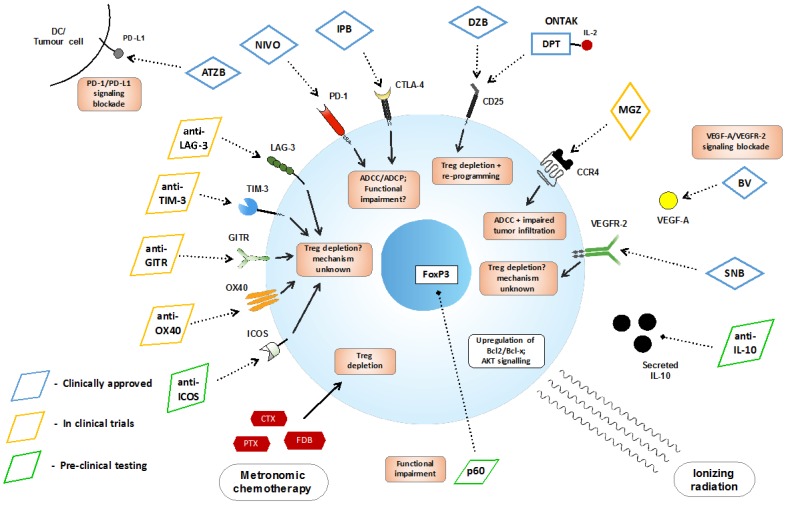
Summary of currently approved and experimental therapies that may target Tregs; therapies are color-coded according to stage in clinical testing. Abbreviations—ADCC: antibody-dependent cell-mediated cytotoxicity, ADCP: antibody-dependent cellular phagocytosis, ATZB: atezolizumab, BV: bevacizumab, CTX: cyclophosphamide, DC: dendritic cell, DZB: daclizumab, CCR4: C-C motif chemokine receptor 4, DPT: diptheria toxin, FDB: fludarabine, IPB: ipilimumab, MGZ: mogamulizumab, NIVO: nivolumab, ONTAK: denileukin difitox, PTX: paclitaxel, SNB: sunitinib.

Given their roles in promoting tumor progression and immune escape, Tregs offer promising therapeutic targets. A number of approaches have been developed and are currently in development to deplete Tregs or impair their suppressive functionality [122,124], as shown in Table 1/Figure 1.

The effect of chemotherapy on Tregs is fairly well-established; low-dose metronomic cyclophosphamide (CTX) can reduce levels of Tregs within TME, TDLN and peripheral blood [125,126]. Treg depletion by low-dose chemotherapy or CD25 blockade prior to adoptive cell therapies, cancer vaccination or other treatment modalities significantly enhances patient survival and development of an effective anti-tumor immune response, as reported in different human cancers [27,28,127,128]. We will focus here on the impact and utility of immune checkpoint inhibition and chemo/radiotherapies for targeting Tregs.

### 3.1. Immune Checkpoint Inhibition and Tregs

Immune checkpoint inhibitors (ICI) for cancer treatment aim to re-establish anti-tumor immune responses by blocking inhibitory immune checkpoint molecules or their ligands, which are often over-expressed on intra-tumoral lymphocyte populations and tumor tissues [52]. ICI strategies have seen significant successes in pre-clinical and clinical trials of non-small cell lung cancers (NSCLC), RCC and melanoma, inducing tumor regression and remission-free survival, although responses are limited to 10%–20% of patients [129,130,131,132]. Monoclonal antibodies (mAbs) against CTLA-4 (ipilimumab) and PD-1 (nivolumab/pembrolizumab) have been approved by the FDA for the treatment of metastatic melanoma, NSCLC, advanced RCC and Hodgkin’s lymphoma.

Despite clinical successes to-date, the exact mechanisms of action of ICI are not fully understood. ICI strategies were initially developed to enhance Teff and CTL functionality. However, it is becoming apparent that ICI might also impact other aspects of the immune system, including depleting or functionally impairing Tregs. As discussed earlier, tumor-infiltrating Tregs highly upregulate expression of various immune checkpoint molecules (CTLA-4, PD-1, LAG-3, TIM-3, GITR), making them viable targets for ICI.

#### 3.1.1. Anti-CTLA-4

The primary proposed mechanism of action of anti-CTLA-4 mAbs is via promotion of T-cell proliferation and activation, due to blockade of CTLA-4/CD28 interactions. Ipilimumab and tremelimumab are two well-characterized IgG1 and IgG2 anti-CTLA-4 mAbs, respectively. Numerous studies report that treatment with ipilimumab or tremelimumab, as monotherapies or in combination with other treatment regimens, induces significant expansion and activation of Teff and CTLs [66,133,134,135,136,137,138,139,140,141]. Interestingly, FoxP3^+^ Tregs have been shown to be stably maintained or increased in the peripheral blood or TILs of cancer patients treated with ipilimumab (Table 2) or tremelimumab (Table 3).

In metastatic prostate cancer and advanced melanoma patients, ipilimumab treatment induced a significant increase in Ag-specific humoral and CTL responses, while levels of suppressive FoxP3^+^ Tregs remained stable or were expanded [66,139,141]. Similarly, in advanced melanoma or RCC patients, treatment with tremelimumab induced broad expansion and activation of CD4^+^ and CD8^+^ T cell subsets, including suppressive Tregs [133]. These studies suggest that CTLA-4 blockade expands and activates Tregs in a similar manner to CTLs and Teff, without affecting their suppressive activity. Indeed, in ipilimumab-treated metastatic prostate cancer patients, almost 50% of circulating FoxP3^+^ Tregs expressed the proliferation marker Ki67 indicating they were proliferating efficiently in vivo [66]. This raises an interesting question regarding ICI and the role of CTLA-4 on Tregs: how can ICI induce tumor regression and anti-tumor immune responses, if both Tregs and Teff are expanded and activated during treatment?

Pre-clinical murine models have shown that, in conjunction with activating Teff and CTLs, anti-CTLA-4 mAbs exert anti-tumor activity via antibody-dependent cell-mediated cytotoxicity (ADCC)-mediated depletion of intra-tumoral Tregs [142,143]. This has recently been reported in human studies [144,145]. Ipilimumab was required for NK cell-mediated ADCC and in vitro depletion of CTLA-4^+^ TI Tregs isolated from TILs of HNSCC patients [144]. Similarly, in a clinical study of melanoma patients, ipilimumab was required for Fc gamma receptor III (FcγRIII)-expressing non-classical monocytes to mediate ADCC of Tregs in vitro [145]. Interestingly, metastatic lesions of melanoma patients who responded to ipilimumab were enriched for FcγRIII-expressing CD68^+^ “inflammatory” macrophages prior to treatment, while intra-tumoral FoxP3^+^ Tregs were depleted following treatment. These preliminary data suggest that ADCC-mediated Treg depletion, and the presence of innate immune cells to mediate ADCC, are important for ICI activity.

An important point to consider is that the immune profile of the peripheral blood does not accurately reflect the TME, and has varying utility as a prognostic factor. In contrast, the make-up of the TME is directly relevant to clinical outcomes. Perhaps the TME of clinical responders is enriched both for CTLA-4^+^ Tregs and FcγRIII-expressing innate immune cells or NK cells. This promotes ADCC-mediated Treg depletion and re-establishment of an anti-tumor immune response following CTLA-4 blockade. Tregs constitutively express CTLA-4, both intra-cellularly and on their surface. The enhanced expression of CTLA-4 may make Tregs more prone to ADCC, and might also explain why Teff and CTLs are not depleted during CTLA-4 blockade.

Tremlimumab, as an IgG2 isotype mAb, is less likely to form immune complexes and induce ADCC compared with ipilimumab, an IgG1 isotype mAb. However, tremelimumab is able to abrogate the suppressive activity of healthy donor Tregs in vitro [138,146]. Treatment with tremelimumab in advanced melanoma patients can selectively confer to PBMCs resistance against Treg-mediated inhibition in in vitro suppression assays. Following treatment, circulating Tregs remained functionally suppressive, and generation of Treg resistance was associated with improved progression-free survival [135].

#### 3.1.2. Anti-PD-1

PD-1 is highly upregulated on “exhausted” T cells, inhibiting T cell proliferation, IFN-γ and IL-2 production [52]. The primary effect of PD-1 blockade is reversal of T cell “exhaustion”. In vitro assays show that nivolumab is able to abrogate Treg suppressive function although it is not clear whether PD-1 blockade acts directly on Tregs or via activation of Teff [151,152]. Nivolumab promoted CTL proliferation and resistance to Treg-mediated suppression, and also impaired Treg suppressive activity, possibly by downregulating intracellular expression of FoxP3 [151]. In metastatic melanoma, combination treatment of nivolumab with a therapeutic peptide vaccine expanded Tregs in the peripheral blood of non-responders. In clinical responders, however, Tregs were depleted over a 12-week treatment period [153,154]. Clinical studies investigating the impact of PD-1 blockade on Tregs are limited (Table 4).

mAbs targeting other immune checkpoints are currently in development or clinical testing, including combination therapies with ipilimumab and nivolumab [129,155,156]. Other targets include the stimulatory immune checkpoints (GITR, OX-40, 4-1BB) and inhibitory immune checkpoints (LAG-3, TIM-3).

### 3.2. Effect of Radiotherapy and Chemotherapy on Tregs

Radiotherapy (RT) adversely affects the immune system, causing severe immune suppression due to non-specific targeting of lymphocytes and haematopoietic progenitor cells. Recent evidence indicates a more complex immune modulating effect of RT, and a potential role for Tregs in determining RT efficacy [157,158].

Tregs are thought to be relatively “radio-resistant”, exhibiting reduced apoptotic potential and increased in vivo proliferation compared to other lymphocyte subsets in response to ionizing radiation [159]. In clinical studies of HNSCC, cervical cancer and glioblastoma multiforme, combination chemo-radiotherapy (CRT) depleted CD4^+^ and CD8^+^ Teff in the peripheral blood and TDLN of patients, while highly suppressive FoxP3^+^ Tregs were unaffected or expanded [160,161,162]. The exact mechanisms of RT-resistance in Tregs have not yet been confirmed in humans, although it may rely on upregulation of Akt signaling and the pro-survival proteins Bcl-2/Bcl-x. T cell activation status also confers radio-resistance. Within the TME, highly suppressive and activated Tregs may exhibit stronger RT-resistance compared to “exhausted” CTLs and naïve T cells. RT also induces the generation of tolerogenic DCs following immunogenic cancer cell death [163].

Given this phenomenon of RT-induced Treg expansion or survival, a number of recent trials are testing RT in combination with ICI or neoadjuvant chemotherapy to deplete Tregs [27,164,165,166]. Results from a pilot study of RT and ipilimumab induced a partial response in only 18% of metastatic melanoma patients [166]. Targeted radio-immunotherapies utilizing radio-labelled anti-CD25 also showed promising results in a clinical trial of non-Hodgkin’s lymphoma [167].

Dosage and timing of RT are critical considerations; low-dose RT may induce anti-inflammatory effects, while higher RT doses deplete Teff and CTLs promoting immune suppression [158,160]. Further insights into the radiobiology of human Tregs and other immune cell subsets will help to improve RT design, taking into account the role of the immune system in clinical outcomes.

## 4. Challenges of Targeting Tregs in Humans

Learning from current efforts to deplete or impair functions of Tregs, there are a number of challenges to consider when designing Treg-targeted therapies in the context of cancer, and more broadly. These are:

### 4.1. Misclassification of Tregs

Accurate and specific Treg markers are essential for two reasons. First, they enable selective targeting of Tregs in vivo without affecting tumor-specific CTLs and Teff. Second, they allow isolation and monitoring of Tregs during treatment for further investigations.

Most clinical trials currently utilize the canonical Treg markers—FoxP3 and CD25—to identify suppressive Tregs. As discussed earlier, FoxP3 and CD25 are also upregulated on activated non-suppressive T cell subsets. A recent consensus meeting proposed an “essential marker set” for suppressive Tregs; CD3^+^/4^+^/25^+^/127^−^ and FoxP3^+^ [102]. Robust Treg marker sets or functional assays must be used to confirm the suppressive lineage of proposed Tregs populations.

### 4.2. Systemic versus Specific Subset Depletion

Understanding the most relevant Treg mechanisms or markers within the TME is important for “rationally designing” Treg-targeted immunotherapies. Systemic depletion of FoxP3^+^ Tregs may result in serious immune-related adverse events. It is imperative to target the most suppressive Treg subsets such as Helios-expressing Tregs [104] and CD45RA^−^FoxP3^hi^CD25^hi^ eTregs [8,64]. In solid tumors, TI Tregs—as suppressive cells on the “front-line”—are the most important Tregs to deplete. Different cancers may have different immune profiles according to location and stage, which can recruit and expand different Treg subsets. For example, mAbs targeting CCR4, a chemokine receptor heavily involved in Treg recruitment to the TME, have shown promising results in clinical and laboratory studies, effectively depleting activated FoxP3^+^CCR4^+^ Tregs with only a limited impact on tumor-infiltrating Teff and CTL subsets [41,45,67,68,69,70].

### 4.3. Tregs in the Immune Context

Neoadjuvant therapies aimed at tipping the balance of immune suppression and immune stimulation in favor of an anti-tumor immune response have shown efficacy in a number of clinical studies [168,169]. Considering clinical outcomes, the key determining factor of clinical benefit in ICI and other therapeutic approaches seems to be the CTL: Treg ratio. CD8^+^ T-cell expansion is associated with better survival in cancer patients treated with anti-CTLA-4 or anti-PD-1 [170,171,172]. For therapeutic success, it is critical that not only Tregs are depleted, but CTLs are released from T cell exhaustion. This might require use of adjuvant therapies to “reinvigorate CTLs”.

## 5. Conclusions

Recent years have highlighted tumor heterogeneity as a critical contributing factor to variable clinical outcomes. Similarly, the immune landscape varies significantly between cancers and patients, influencing the role of Treg subsets in cancer; whether as part of an immunosuppressive network promoting tumor immunity or a protective mechanism controlling cancer-associated inflammation. Tumor-infiltrating Tregs are “front-line” players in the immune response and cancer. From a biological standpoint, understanding the role and contribution of diverse Treg subsets in the tumor microenvironment is a critical first step to designing better therapies. Clinically, the advent of immune checkpoint inhibition, in addition to currently available therapies, offer useful treatment paradigms for Treg depletion or impairment. Targeting tumor-infiltrating Tregs—likely as part of a multi-modal treatment strategy—offers an exciting treatment paradigm to re-establish anti-tumor immunity and break immunosuppressive networks within the tumor microenvironment. As discussed here, translating our current knowledge of Treg immunobiology into viable immunotherapies requires a deeper understanding of the immune dynamics of Tregs in various malignancies, the interactions of Tregs with tumor-infiltrating cell subsets, and the impact of different therapeutic modalities on Tregs in vivo.

## Figures and Tables

**Table 1:** Therapeutic modalities for targeting Tregs (reviewed in [124]).

Therapy	Modality
Low-dose chemotherapy	Treg depletion
CD25-targetted Abs	Treg depletion
Immune checkpoint inhibition (ICI)	Functional targeting + Treg depletion
Chemokine receptor blockade	Functional targeting + Treg depletion
Blockade of suppressive mechanisms & soluble mediators (IL-10/TGF-β)	Functional targeting

**Table 2:** Clinical studies investigating the impact of ipilimumab on Tregs.

Cancer	Treg Markers	PB/TILs	Functional Analysis	Expanded?	Survival	Ref.
Resected stage IIIc/IV melanoma (*n* = 75)	CD25^+^	PB	Suppressive; no effect after treatment	No change	N/A	[147]
Unresectable stage III/IV melanoma (*n* = 80)	CD25^hi^CD127^lo^FoxP3^+^	PB	N/A	No change at weeks 4 & 12	N/A	[139]
Stage IV malignant melanoma	CD25^+^FoxP3^+^	PB	N/A	Decreased	No statistical link	[148]
Bladder cancer patients prior to radical cystectomy (*n* = 6)	CD25^+^FoxP3^+^	PB	Suppressive pre/post- treatment	Overall decrease; variable initial response	N/A	[149]
Bladder cancer patients prior to radical cystectomy (*n* = 6)	CD25^+^FoxP3^+^	TILs	NA	Increase in ICOS+ Teff : FoxP3+ Treg ratio	N/A	[149]
Bladder cancer (*n* = 12)	CD25^+^LAP^+^/FoxP3^+^/CD127^lo^	PB	CD25^+^LAP^+^, but not CD25^+^CD127^lo^, suppressive post-treatment	CD25^+^LAP^+^ increased in patient subset	N/A	[55]
Metastatic RCC or metastatic melanoma (*n* = 10)	CD25^+^FoxP3^+^	PB	Suppressive pre/post-treatment	No change; increase in activated T cells	N/A	[133]
Progressive metastatic hormone-refractory prostate cancer (*n* = 24)	CD127^lo^CD25^hi^	PB	Suppressive post-treatment	Increased, and Ki67^+^	N/A (study* ongoing)	[66]
Stage III/IV melanoma (*n* = 37)	CD25^HI^Foxp3⁺	PB	N/A	Increased at 6 weeks post-treatment	Associated with improved PFS	[141]
Stage III/IV melanoma (*n* = 10)	CD25^HI^Foxp3⁺	TILs	N/A	Variable	Inverse trend between Treg & clinical benefit	[141]
Stage IV melanoma (*n* = 82)	CD127^lo^CD25^+^FoxP3^+^	PB	N/A	Increased over 14 weeks of treatment	Higher than median Tregs associated with better survival	[150]

All studies utilized CD4 as a T cell marker and dosage is 1–10 mg/kg; N/A: Not investigated; RCC: renal cell carcinoma; PB: peripheral blood; TILs: tumor-infiltrating lymphocytes; * Clinical trial identifier: NCT00064129.

**Table 3:** Clinical studies investigating the impact of tremelimumab on Tregs.

Cancer	Treg marker	PB/TILs	Functional Analysis	Expanded?	Survival	Ref.
DTIC-treated stage IV melanoma (*n* = 10)	CD25^+^CD127^−^ or FoxP3^+^	PB	Suppressive pre-treatment; transient resistance to Treg suppression post-treatment	Increase in absolute Treg count, but not proportion	Treatment-induced transient Treg resistance associated with better survival	[135]
Stage III/IV melanoma, combined with IFN-α2b (*n* = 37)	CD25^hi^FoxP3^+^ or CD25^hi^CD39^+^	PB	N/A	Both subsets Increased	N/A	[140]
Metastatic melanoma (*n* = 7)	CD25^+^FoxP3^+^	TILs	N/A	Variable	N/A	[136]

All studies utilized CD4 as a T cell marker unless state otherwise; dosage is 1–15 mg/kg; N/A: Not investigated; IFN-α2b: interferon alfa 2b anti-viral drug; PB: peripheral blood; TILs: tumor-infiltrating lymphocytes.

**Table 4:** Clinical studies investigating the impact of nivolumab on Tregs.

Cancer	Treg Markers	PB/TILs	Functional Analysis	Expanded?	Survival	Ref.
Unresectable stage III/IV melanoma (*n* = 90); IPB-naive (*n* = 34) or IPB-refractory (*n* = 56)	CD25^+^CD127^lo^FoxP3^+^	PB	N/A	Decreased in responders & stable patients; increased in non-responders	Increased Tregs associated with progression at 12 weeks	[153]
Stage IIIc/IV melanoma (*n* = 33)	CD127^lo^FoxP3^+^	PB	N/A	Expanded in PB at 12 & 24 weeks	Trend towards lower Tregs in non-relapsing patients	[154]

All studies utilized CD4 as a T cell marker; dosage is 1–10 mg/kg; N/A: Not investigated; IPB: Ipilimumab; PB: peripheral blood; TILs: tumor-infiltrating lymphocytes.

## Abbreviations

The following abbreviations are used in this manuscript:

Ab: antibody
ADCC: antibody-dependent cell-mediated cytotoxicity
ADCP: antibody-dependent cellular phagocytosis
Ag: antigen
APC: antigen presenting cell
CCR: C-C motif chemokine receptor
CD25: IL-2 receptor alpha chain
COX-2: cyclooxygenase-2
CRC: colorectal cancer
CRT: chemo-radiotherapy
CTL: CD8+ cytotoxic t cell
CTLA-4: cytotoxic T-lymphocyte-associated protein 4
CTX: cyclophosphamide
CXCR: CXC chemokine receptor
eTreg: effector Treg
FcγRIII: Fc gamma receptor III
FoxP3: forkhead box P3
GARP: glycoprotein A repetitions predominant
GITR: glucocorticoid-induced TNFR-related protein
HAVCR2: Hepatitis A virus cellular receptor 2
HBV/HCV: hepatitis B/C virus
HCC: hepatocellular carcinoma
HNC: head and neck cancer
HNSCC: head and neck squamous cell carcinoma
HPV: human papilloma virus
ICI: immune checkpoint inhibition
ICOS: Inducible T-cell costimulator
IDO: indoleamine 2,3-dioxygenase
IFN-γ: interferon gamma
IL-10/35: Interleukin 10/35
LAG-3: lymphocyte activation gene-3
LAP: latency-associated peptide
mAb: monoclonal antibody
MDSC: myeloid-derived suppressor cell
NK cell: natural killer cell
NRP1: neuropilin 1
PD-1: programmed death 1
PD-L1: programmed death-ligand 1
PGE-2: prostaglandin E-2
pTreg: peripheral Treg
RANK: receptor activator of nuclear factor kappa-B
RANKL: receptor activator of nuclear factor kappa-B ligand
RCC: renal cell carcinoma
RT: radiotherapy
S1P: sphingosine-1-phosphate
S1PR1: sphingosine-1-phosphate receptor 1
TAA: tumor-associated antigen
Tconv: conventional T cell
TDLN: tumor-draining lymph node
Teff: CD4+ T effector cell
TEX: tumor-derived exosome
TGF-β: Transforming growth factor beta
Th cell: T helper cell
TI: tumor-infiltrating
TIGIT: T cell immunoreceptor with Ig and ITIM domains
TILs: tumor-infiltrating lymphocytes
TIM-3: T-cell immunoglobulin and mucin-domain containing-3
TME: tumor microenvironment
Treg: regulatory T cell
tTreg: thymic-derived Treg
VEGF-A: vascular endothelial growth factor-A
γδ T cell: gamma delta T cell
